# Community‐based health insurance service utilization and associated factors in Addis Ababa, Ethiopia

**DOI:** 10.1002/puh2.18

**Published:** 2022-08-20

**Authors:** Aderajew Mekonnen Girmay, Mulumebet Tadesse Reta

**Affiliations:** ^1^ Ethiopian Public Health Institute Addis Ababa Ethiopia; ^2^ Gullele Sub City Health Office Addis Ababa Ethiopia

**Keywords:** community, fee waiver, health‐insurance, quality service, utilization

## Abstract

**Introduction:**

Community‐based health insurance (CBHI) scheme is a powerful tool to achieve universal health service coverage by providing financial protection against healthcare costs. Ethiopia introduced CBHI, although its utilization is suspected to be poor. Consequently, this study aimed to assess CBHI service usage and related factors.

**Methods:**

Six hundred fifty‐two participants were enrolled in this study. Data was collected through a face‐to‐face interview.

**Results:**

This study indicated that 60% of respondents utilized the CBHI service. In this study, male participants were 44% less likely to use the CBHI service (AOR = 0.44 with 95%CI: 0.28–0.70) than female participants. Besides, participants from small households were 54% less likely to utilize the CBHI service (AOR = 0.54 with 95%CI: 0.36–0. 82) than those from large families. However, using the CBHI service was 4.97 times higher among illiterate participants (AOR = 4.97 with 95%CI: 1.46–16.91) than those with literacy skills. Moreover, participants with low income were 5.14 times more likely to use the CBHI service (AOR = 5.14 with 95%CI: 2.27–11.64) than those with high monthly income. Additionally, participants who had not a private home were 6.31 times higher to use the CBHI service (AOR = 6.31 with a 95%CI: 2.76–14.46) than those who had. Furthermore, participants having adequate information on the CBHI were 8.99 times more likely to use the CBHI service (AOR = 8.99 with 95%CI: 5.66–14.27) than those who had not.

**Conclusion:**

The study provides essential information regarding CBHI service utilization and associated factors. Although the majority of the participants used the CBHI service, still a large number of users did not receive the services properly. Availability of laboratory services and delivery of essential drugs were low. Therefore, government and other relevant agencies should develop effective regulatory systems to promote the CBHI service and improve public health facilities' capacity to provide CHHI services properly.

## INTRODUCTION

Community‐based health insurance (CBHI) scheme is a powerful tool to achieve universal health service coverage by providing financial protection against healthcare costs [1]. Ethiopia introduced CBHI, although its utilization is suspected to be poor. Consequently, this study aimed to assess CBHI service usage and related factors. The CBHI is one of the solutions proposed by the World Health Organization (WHO) to improve universal health coverage. Because worldwide, no country can fully provide health insurance to citizens effectively due to a lack of spending money for health care services [2]. The WHO emphasizes universal health coverage as 150 million people worldwide suffer from a financial catastrophe yearly [3]. Additionally, more than 100 million are pushed into poverty due to direct payments for healthcare‐related services [3]. Even in the developed countries such as the United State America (USA) could not offer health service cover to all citizens, and 46 million Americans have insufficient health services coverage [4].

Although equity to health service is the underlying principle of major global public health strategies such as the health sector reforms spearheaded by the World Bank and the current Sustainable Development Goals (SDGs) 3, its implementation is a significant public health challenge [3, 5]. Many studies revealed that several low‐income countries are challenged to sustain healthcare financing [6, 7]. However, achieving universal health coverage, including financial risk protection, access to quality healthcare services, and access to safe, adequate, quality, and affordable essential medicines are fundamental for sustaining growth and development [8]. Mainly, enhancing health outcomes and protecting households against the financial consequences of ill health are the highest priorities to reduce poverty and sustain growth [9]. However, in developing countries, many people are suffering and dying due to a lack of access to health services [10]. This could be mainly due to the inability of the poor to pay for healthcare services. A study covering about 89 per cent of the world's population suggests that 150 million people suffer from a financial catastrophe yearly [11]. Specifically, over 90% of healthcare financial difficulties and their consequences occurred in Sub‐Saharan African countries where resources are limited [12, 13]. Many studies indicated that the health insurance cover and uptake is low in sub‐Saharan Africa [14, 15]. Therefore, establishing an effective and responsive health delivery system in low‐income countries, including Ethiopia, is an integral part of the overall development that aims to reduce poverty and achieve SDGs [16]. Though Ethiopian health system provision has three tiers: primary care level, secondary care, and tertiary levels [17], the country is one of the highest disease burden countries where the utilization of facility‐based healthcare is very low [18].

Notably, the poor individual citizens of the country are often suspected of declining care when it is needed. Because they cannot meet the expense of health service costs, due to this and other unknown factors they may be forced to take self‐administered local medicines and go to traditional healers, which could pose additional health risks. To rectify the above problems and achieve universal health coverage, the government of Ethiopia introduced the CBHI to protect the eligible poor and other vulnerable groups [19]. This is a user fee payment waiver program designed to improve the health of poor citizens [20, 21]. This fee waiver system targets a person who cannot afford to pay a user fee for health services [22, 23]. Despite the CBHI scheme providing significant improvements in the health of the poor and vulnerable group there are a lot of challenges regarding the utilization of CBHI. Remarkably, the public health facilities of the Addis Ababa city administration are suspected of having several challenges, including shortage of drugs, absence of diagnostic and therapeutic procedures, poor patient handling practice, bureaucratic process, lack of treatment availability, and poor‐quality health service provision to the CBHI service users. Therefore, ascertaining and closing this gap requires research. Additionally, scientific evidence is necessary since nothing is known about the current status of CBHI service utilization and determinant factors in Addis Ababa city administration. Hence, this study aimed to assess the level of CBHI service utilization and identify core determinant factors that affect the service.

## METHODS

### Study design, study population, and study area

A community‐based cross‐sectional study was conducted in the community of Gullele sub‐city from September to November 2021. All CBHI beneficiary households were the source population. All selected CBHI beneficiary heads of families who live in the sub‐city were the study participants. In this study, all head of households who were CBHI beneficiaries and had encountered health problem in the past year was the inclusion criteria. However, heads of households who had mental and severe illnesses were excluded from the study. The study was conducted in Addis Ababa city Administration Gullele Sub‐city. The sub‐city is organized into 10 districts. To provide preventive and curative services, it has two referral hospitals, 10 public health centers, 10 district health offices, 48 private clinics, and two None‐Governmental Organization (NGO) health centers. According to the Gullele health office 2021 report, the sub‐city has more than 21,000 CBHI users.

### Sampling size determination, sampling method, and data collection procedures

The sample size was determined using a single population proportion formula with the assumptions of a 95% confidence interval (*α* = 0.05), 5% margin of error, population proportion of CBHI service utilization (74%) [24], two design effect, and 10% for the non‐respondent rate. Based on this, six hundred fifty‐two participants were enrolled in this study. The study participants were selected using a stratified, simple random sampling technique. In the first stage, a listing of the existing CBHI service users was obtained from the Gullele sub‐city health office. Users were stratified into 10 administrative districts. Then, the heads of households who have encountered health problems in the past year were registered. Using a proportional sample allocation formula, sample allocation was done to each district based on the number of written heads of households. Finally, study subjects were selected using a simple random sampling method. Data was collected through a face‐to‐face interview. A questionnaire was prepared in English and translated into Amharic and back to English to keep the consistency of the questions. Data quality was ensured through data collectors’ training, close supervision, prompt feedback, and daily rechecks of the completed questionnaire.

### Study variables

To determine the CBHI service utilization respondents were asked one Yes/No question whether they used the CBHI service at the time of illness at least once in the past year. The outcome variable of the study was CBHI service utilization. However, predictor variables of the study were age, sex, educational status, marital status, occupation, family size, monthly income, having a private home, prevalence of non‐communicable diseases, health service utilization status, quality healthcare service, availability of laboratory service, and availability of drug supply, using all essential treatments, health professional care, using private health facilities services, having adequate information about CBHI, bureaucratic function and delay in obtaining the CBHI service.

### Data analysis

The data was checked for the correctness of information and code. Data analyses were performed using SPSS (Statistical Package for the Social Sciences) software version 20. Moreover, binary logistic regression and multivariable regression analyses were used to analyze the data. In all analyses, *p*‐value < .05 was considered statistically significant.

### Ethical consideration

Ethical approval was obtained from the Institutional Review Board (IRB) of the Addis Ababa Health Bureau with reference number AAHB/9566/12/21. Written consent was obtained from the study subjects. All the rights of the study subjects were clearly stated in the questionnaire.

## RESULTS

### Socio‐demographic characteristics of respondents

Of the 652 heads of households, 648 consented to the study producing a 99.4% response rate. In this study, above three‐fourths (77.6%) of the participants were female. Additionally, 30.6% and 26.4% of the respondents were between the age group 56–65 and 46–55, respectively. The mean age of the respondents was 55.67. The majority (58.8%) of respondents were married. Most (92.4%) of the participants had at least literacy skills while 7.6% were illiterate. Nearly half (46.8%) of the respondents were retired and employed in the private sector. Above half (53.2%) of the participants were employed in government and non‐government agencies. Almost two‐thirds (62.2%) of the respondents had less than four family members. The average family size was 4. Most (91.8%) of the respondents had a monthly income of under 3000 Ethiopian Birr or < 83.33 USD. The mean of participants’ income was 1,523.59 Ethiopian Birr or 42.32 USD. The majority (91.7%) of the participants had no private homes (Table 1).

**TABLE 1:** Sociodemographic characteristics of respondents (*n* = 648)

Study variables	Category	Frequency	Percent (%)	Mean	Standard deviation
Sex	Male	145	22.4		
	Female	503	77.6		
Age	15–25 years	10	1.5	55.67	12.703
	26–35 years	35	5.4		
	36–45 years	96	14.8		
	46–55 years	171	26.4		
	56–65 years	198	30.6		
	>65 years	138	21.3		
Marital status	Single	51	7.9		
	Married	381	58.8		
	Divorced	96	14.8		
	Widowed	120	18.5		
Education level	Illiterate	49	7.6		
	At least reading and writing	599	92.4		
Occupation	Retired and private work	303	46.8		
	Governmental and NGO employed	345	53.2		
Family size	<4 member	403	62.2	4.13	1.73
	≥4 member	245	37.8		
Monthly income	<3000 Birr (<83.33 USD)	595	91.8	1523.59	1170.29
	≥3000 Birr (≥83.33 USD)	53	8.2		
Having a private home	No	594	91.7		
	Yes	54	8.3		

### Prevalence of chronic diseases among the CBHI members

In this study, 8.6% and 8.2% of respondents had known hypertension and diabetes mellitus, respectively. Additionally, 1.4% and 4.2 of the participants had known cardiovascular diseases and asthma, kidney and epilepsy, respectively (Table 2).

**TABLE 2:** Prevalence of non‐communicable diseases among respondents (*n* = 648)

Study variables (Type of chronic disease)	Category	Frequency	Percent (%)
Having hypertension	Yes	56	8.6
	No	592	91.4
Having diabetes mellitus	Yes	53	8.2
	No	595	91.8
Having heart case (cardiovascular disease)	Yes	9	1.4
	No	639	98.6
Having asthma, kidney and epilepsy	Yes	27	4.2
	No	621	95.8

### Health service utilization among the CBHI members

This study indicated that 60% of respondents utilized the CBHI service. Moreover, 86.4% of participants used any health service in the past year. In this study, 38% of respondents were obtained all laboratory services. However, 62% of respondents did not receive laboratory services. In addition, 34.1% of participants used all essential drugs in the government healthcare. However, 65.9% of respondents did not get essential drugs in the government healthcare. Also, 33.6%, 49.5%, and 27.9% of respondents used all essential treatments from the governmental healthcare settings, obtained good health professional approach in the governmental health institutions, and used private health facilities services once or more time after being a member of the CBHI, respectively. However, 66.4% and 50.5% of the eligible service users were not used all essential treatments and did not obtain good health professional care in the government health institutions, respectively. In this study, 52.6% and 35% of respondents were not obtain adequate information about the advantage of the CBHI from government media and they faced bureaucratic functions and delays in getting the CBHI service in the governmental healthcare settings, respectively (Table 3).

**TABLE 3:** Health service utilization among the CBHI members

Study variables	Category	Frequency	Percent (%)
Any health service utilization in the past year	Yes	560	86.4
	No	88	13.6
CBHI service utilization at least once in the past year	Yes	389	60.0
	No	259	40.0
Received quality healthcare service	Yes	269	41.5
	No	379	58.5
Obtained all laboratory services	Yes	246	38.0
	No	402	62.0
Obtained all essential drugs	Yes	221	34.1
	No	427	65.9
Utilized all essential treatments	Yes	218	33.6
	No	430	66.4
Obtained good health professional care	Yes	321	49.5
	No	327	50.5
Used private health facilities services once or more time after being a member of the CBHI	Yes	181	27.9
	No	467	72.1
Having adequate information about CBHI	Yes	307	47.4
	No	341	52.6
Bureaucratic function and delay in obtaining the CBHI service	Yes	227	35.0
	No	421	65.0

### Binary logistic regression analysis

In the binary logistic regression analysis, 10 predictor variables, including sex, age group, education level, occupation, family size, monthly income, having a private home, using private health facilities services, having adequate information about CBHI, bureaucratic function and delay to obtain the CBHI service were significantly associated (*p*‐value < 0.05) with the CBHI service utilization. In the binary logistic regression analysis, participants having age 60 and above years (retired) were 2.02 times higher to use the CBHI services than those having less than 60 years old (Crude Odd Ratio (COR) = 2.02 with 95%CI: 1.44–2.83). Besides, the odds of respondents having retired and private work had 2.04 times higher to use the CBHI services than those who had working in government and NGO (COR = 2.04 with 95%CI: 1.48–2.81). However, respondents who used services of private healthcare facilities were 31% less likely to use the CBHI service (COR = 0.31 with 95%CI: 0.22–0.45) (Table 4).

**TABLE 4:** Binary logistic regression analysis (*n* = 648)

		CBHI service utilization					
Study variables		Yes	No	Β	Wald	*p*‐Value	COR with 95%CI
Sex	Male	67	78	‐0.73	14.57	0.000	0.48 (0.33‐0.70)
	Female	322	181				Reference
Age group	>60 years (retire)	172	73	0.70	16.75	.000	2.02 (1.44‐2.83)
	≤60 years	217	186				Reference
Educational level	Illiterate	44	5	1.87	15.21	0.000	6.48 (2.53‐16.57)
	At least reading and writing	345	254				Reference
Occupation	Retired and private work	209	94	0.71	18.75	0.000	2.04 (1.48‐2.81)
	Government and non‐government employed	180	165				Reference
Family size	<4 members	224	179	‐0.50	8.73	0.003	0.61 (0.44‐0.85)
	≥4 members	165	80				Reference
Monthly income	<3000 Birr (<83.33 USD)	376	219	1.66	25.38	0.000	5.28 (2.77‐10.09)
	≥3000 Birr (≥83.33 USD)	13	40				Reference
Having a private home	No	376	218	1.69	26.43	0.000	5.44 (2.85‐10.38)
	Yes	13	41				Reference
Utilized private health facilities services once or more times after being a member of the CBHI	No	72	109	‐1.16	41.13	0.000	0.31 (0.22‐0.45)
	Yes	317	150				Reference
Having adequate information about CBHI	Yes	243	64	1.62	83.10	0.000	5.07 (3.58‐7.19)
	No	146	195				Reference
Bureaucratic function and delay in obtaining the CBHI service	Yes	96	131	‐1.14	44.31	0.000	0.32 (0.23‐0.45
	No	293	128				Reference

### Multivariable logistic regression analysis

In this model, only seven predictor variables, including sex, education level, family size, monthly income, having a private house, having adequate information about CBHI, presence of bureaucratic function and delay in obtaining the CBHI service were significantly associated (*p*‐value < 0.05 at 95%CI) with the CBHI service utilization (Table 5).

**TABLE 5:** Multivariable logistic regression analysis (*n* = 648)

		Community‐based health insurance service utilization					
Study variables		Yes	No	Β	Wald	*p*‐Value	Adjusted odd ratio (AOR) with 95%CI
Sex	Male	67	78	‐0. 82	11.90	0.001	0.44 (0. 28‐0.70)
	Female	322	181				Reference
Education level	Illiterate	44	5	1.60	6.61	0.01	4.97 (1.46‐16.91)
	At least reading and writing	345	254				Reference
Family size	<4	224	179	‐0.61	8.47	0.004	0.54 (0.36‐0.82)
	≥4	165	80				Reference
Monthly income	<3000 Birr (< 83.33 USD)	376	219	1.64	15.42	0.000	5.14 (2.27‐11.64)
	≥3000 Birr (≥83.33 USD)	13	40				Reference
Having a private house	No	376	218	1.84	19.00	0.000	6.31 (2.76‐14.46)
	Yes	13	41				Reference
Having adequate information about CBHI	Yes	243	64	2.20	86.51	0.000	8.99 (5.66‐14.27)
	No	146	195				Reference
Bureaucratic function and delay in obtaining the CBHI service	Yes	96	131	‐1.16	9.78	0.002	0.32 (0.15‐0.65)
	No	293	128				Reference

## DISCUSSION

CBHI scheme is a powerful tool to achieve universal health service coverage with adequate financial protection against healthcare costs. Hence, assessing its utilization status and determinant factors is a crucial activity. In this study, the self‐reported CBHI service utilization was 60%. This revealed that most respondents utilized the fee waiver privilege properly and saved them from health service expenses. Though the fee waiver system is in its early stage in Addis Ababa, it is good to care for the health of the poor who cannot be able to cover their illness‐associated costs. This finding was higher than a study conducted in southern Ethiopia in which the CBHI service utilization was 39.1% [25]. This might be due to the difference in socioeconomic disparities and urbanization. The study findings indicated that only 38% and 34.1% of the participants were obtained all laboratory services and essential drugs within the governmental healthcare.

On the other hand, 62% and 65.9% of them were exposed to extra expenses for laboratory tests and drugs; since the CBHI did not cover waivers when a patient service is outside government health facilities. Also, this indicated that the availability of laboratory services and drug supply in the public health facility was very low. Poor governmental attention and low participation of relevant agencies could be the main reason.

In this study, only one‐third (33.6%) of respondents obtained all essential treatments in governmental healthcare institutions. This indicated that most health services were unavailable, which may obligate waiver users (CBHI service users) to use privately owned health services. Also, this finding approved the ineffectiveness of the CBHI scheme though it has many advantages for the poor. This finding is consistent with a study done in Kenya [26]. The result of the current study indicated that only 41.5% of the CBHI users had obtained quality service. This finding assured that the provision of quality service by the public health facilities was poor. The main reason could be the lack of skilled professionals and shortage of equipment due to poverty, although this result is in line with a study conducted in Ethiopia [27].

In this study, male participants were 44% less likely to use the CBHI service (AOR = 0.44 with 95%CI: 0.28–0.70) than female participants. The reason could be due to the differences in health‐seeking behavior. Additionally, this could be due to the preference of males to use private health services. Further, participants from small households were 54% less likely to utilize the CBHI service (AOR = 0.54 with 95%CI: 0.36–0. 82) than those from large families. This finding is consistent with a study conducted in Ethiopia [25]. However, using the CBHI service was 4.97 times higher among illiterate participants (AOR = 4.97 with 95%CI: 1.46–16.91) than those with literacy skills. Differences in trust, absence of alternative income, and choice could be the main reasons, although it needs further research. Moreover, participants with low income were 5.14 times more likely to use the CBHI service (AOR = 5.14 with 95%CI: 2.27–11.64) than those with high monthly income. This is expected. As participants' income decreases, the chance of utilizing CBHI services may increase. Additionally, participants who had not a private home were 6.31 times higher to use the CBHI service (AOR = 6.31 with a 95%CI: 2.76–14.46) than those who had. Furthermore, participants having adequate information on the CBHI were 8.99 times more likely to use the CBHI service (AOR = 8.99 with 95%CI: 5.66–14.27) than those who had not. As stated by Sir Francis Bacon, this finding assured that the idea of “information is power” and “The better information one has, the more one will be able to control events.” https://projectools.com/information‐is‐power/

However, respondents who faced bureaucratic function and delay were 0.32 times less likely to use the CBHI service (AOR = 0.32 with 95%CI: 0.15–0.65) compared to those who had not. This may make patients prefer private health institution services and traditional medicines, which may pose additional risk to health.

## LIMITATION OF THE STUDY

Since the study is cross‐sectional, it could not indicate the cause and effect of why the CBHI users did not use it. Furthermore, the study did not show the cause and effect correlations between CBHI service utilization and associated factors. Additionally, the study may have a recall, information, and social desirability bias.

## CONCLUSION

The study provides essential information regarding CBHI service utilization and associated factors. CBHI scheme is a vital tool to improve universal health service coverage and to save the lives of the poor. Although the majority of the participants used the CBHI service, still a large number of users did not receive the services properly. Availability of laboratory services and delivery of essential drugs were low. Only one‐third of the CBHI users used all essential services free of charge. Sex, education level, family size, income, having a private house, having adequate information about CBHI, bureaucratic functions, and delay of patients were significantly associated with the CBHI services utilization. Therefore, government and other relevant agencies should develop effective regulatory systems to promote the CBHI service and improve public health facilities' capacity to provide CHHI services properly. In addition, further research should be undertaken to identify additional factors influencing the utilization of CBHI services.

## CONFLICT OF INTEREST

We, the authors, declare that we have no competing interests.

## FUNDING

This research did not receiveany specific grant from funding agencies in the public, commercial, or not‐for‐profit sectors.

## ETHICAL APPROVAL

Ethical approval was obtained from the institutional review board (IRB) of the Addis Ababa health bureau with reference number AAHB/9566/12/21.

## AUTHOR CONTRIBUTIONS

Aderajew Mekonnen Girmay: Conceptualization; Data curation; Formal analysis; Investigation; Methodology; Project administration; Resources; Software; Supervision; Validation; Visualization; Writing – original draft; Writing – review & editing. Mulumebet Tadesse Retta: Data curation; Funding acquisition; Investigation; Resources; Supervision; Validation; Visualization; Writing – review & editing.

## Data Availability

All data relevant to the study are included in the article.
